# Short- and long-term outcomes of infective endocarditis admission in adults: A population-based registry study in Finland

**DOI:** 10.1371/journal.pone.0254553

**Published:** 2021-07-15

**Authors:** Elina Ahtela, Jarmo Oksi, Tero Vahlberg, Jussi Sipilä, Päivi Rautava, Ville Kytö

**Affiliations:** 1 Heart Center, Turku University Hospital and University of Turku, Turku, Finland; 2 Department of Infectious Diseases, Turku University Hospital and University of Turku, Turku, Finland; 3 Department of Clinical Medicine, Biostatistics, University of Turku and Turku University Hospital, Turku, Finland; 4 Department of Neurology, Siun sote, North Karelia Central Hospital, Joensuu, Finland; 5 Clinical Neurosciences, University of Turku, Turku, Finland; 6 Department of Public Health, University of Turku, Turku, Finland; 7 Turku Clinical Research Centre, Turku University Hospital, Turku, Finland; 8 Research Center of Applied and Preventive Cardiovascular Medicine, University of Turku, Turku, Finland; 9 Center for Population Health Research, Turku University Hospital and University of Turku, Turku, Finland; 10 Administrative Center, Hospital District of Southwest Finland, Turku, Finland; Lundquist Institute at Harbor UCLA Medical Center, UNITED STATES

## Abstract

Infective endocarditis (IE) is associated with high mortality. However, data on factors associated with length of stay (LOS) in hospital due to IE are scarce. In addition, long-term mortality of more than 1 year is inadequately known. In this large population-based study we investigated age and sex differences, temporal trends, and factors affecting the LOS in patients with IE and in-hospital, 1-year, 5-year and 10-year mortality of IE. Data on patients (≥18 years of age) admitted to hospital due to IE in Finland during 2005–2014 were collected retrospectively from nationwide obligatory registries. We included 2166 patients in our study. Of the patients 67.8% were men. Women were older than men (mean age 63.3 vs. 59.5, p<0.001). The median LOS was 20.0 days in men and 18.0 in women, p = 0.015. In the youngest patients (18–39 years) the median LOS was significantly longer than in the oldest patients (≥80 years) (24.0 vs. 16.0 days, p = 0.014). In-hospital mortality was 10% with no difference between men and women. Mortality was 22.7% at 1 year whereas 5- and 10-year mortality was 37.5% and 48.5%, respectively. The 5-year and 10-year mortality was higher in women (HR 1.18, p = 0.034; HR 1.18, p = 0.021). Both in-hospital and long-term mortality increased significantly with aging and comorbidity burden. Both mortality and LOS remained stable over the study period. In conclusion, men had longer hospital stays due to IE compared to women. The 5- and 10-year mortality was higher in women. The mortality of IE or LOS did not change over time.

## Introduction

Infective endocarditis (IE) is a disease associated with high mortality. During the recent years the diagnostics of IE including imaging technology, such as echocardiography and nuclear imaging, have evolved remarkably enabling better diagnostic accuracy [1]. Furthermore, modifications have been made in antibiotic therapy and surgical approach guidelines for IE due to novel research data [1]. Despite the improved diagnostic and treatment methods the mortality of IE has remained unchanged over the last years [2–4]. The short-term mortality (i.e. within 30 days or during the hospital admission) of IE has been reported to range from 10 to 24% [2,3,5,6] and the longer-term mortality (i.e. 6 months to 1 year) from 22 to 37% [6–9]. Similar short-term [5,10,11] and longer-term mortality [9,12,13] between the sexes has previously been described, whereas higher mortality either in women [14,15] or men [16] has also been found. In addition to the older age and comorbidities, *Staphylococcus aureus* etiology has been reported to be a predictor of mortality in IE [4–6,14,17].

The median length of stay (LOS) in the hospital due to IE has been reported to be 7–43 days with a substantial variation between studies from different countries [2,5,8,14,18–20]. Longer hospital admissions have been reported in patients that had surgery [20,21], whereas shorter LOS have been described in patients that died during the hospital stay [5,22]. However, data on factors associated with LOS in IE patients are scarce. In addition, the long-term mortality of more than 1 year is inadequately known. In this large population-based study we investigated age and sex differences, temporal trends, and factors affecting LOS due to IE and in-hospital, 1-year, 5-year and 10-year mortality of IE.

## Materials and methods

We studied adult patients (≥18 years of age) admitted to the hospital due to IE in Finland during 2005–2014. Follow-up ended December 31, 2016, or at 10 years following discharge, whichever came first. Data were collected retrospectively from the nationwide Care Register for Health Care database (CRHC) maintained by the Finnish National Institute for Health and Welfare [23]. This mandatory database automatically collects hospital discharge data including individual baseline data (e.g. age, sex, admission, surgery and discharge dates), discharge diagnoses from the International Classification of Diseases, Tenth revision (ICD-10) and surgical procedure codes (Nordic Classification of Surgical Procedures) of all hospital admissions in Finland. Survival data was obtained from the nationwide, mandatory-by-law Cause of Death Registry held by Statistics Finland. Databases were accessed on July 8, 2018.

Patients discharged from medical or surgical care units of all 38 hospitals (including 5 university hospitals) treating acute IE between January 1, 2005 and December 31, 2014 were included. Surgery of the heart or ascending aorta as well as pacemaker implantations or changes performed within 1 year prior to the admission due to IE during study period were collected from CRHC. The study was approved by the Hospital District of Southwest Finland (permission no. TO2/015/17), National Institute for Health and Welfare (THL/1349/5.05.00/2015) and Statistics Finland (TK53-1410-15). Legal basis for processing of personal data is public interest and scientific research (EU General Data Protection Regulation 2016/679 (GDPR), Article 6(1)(e) and Article 9(2)(j); Data Protection Act, Sections 4 and 6). Patient consent was waived due to the retrospective study design. According to Finnish law, patient consent is not demanded because the retrospective patient data are accessible by law.

We included patients with discharge diagnosis of IE (ICD-10 codes I33, I38 and I39) as primary (67.6%), secondary (23.4%) or tertiary (9.0%) cause of admission. Specificity of these ICD-10 IE codes was previously studied in a subgroup of patients admitted to the Turku University Hospital. Patient data (including e.g. laboratory, microbiology, pathology and imaging data) of randomly selected patients (n = 188; 74% male, mean age 59.7 years) admitted during 2005–2014 were investigated to determine whether the modified Duke criteria for IE [1,24] were fulfilled. Of 188 evaluated patients, 182 fulfilled the criteria (definitive IE in 122 and possible IE in 60 patients) resulting to specificity of 96.8% [23].

Charlson Comorbidity Index (CCI) score including baseline diabetes mellitus, congestive heart failure, peripheral vascular disease, myocardial infarction, cerebrovascular disease, dementia, chronic pulmonary disease, rheumatic disease, peptic ulcer disease, liver disease, hemi- or paraplegia, renal disease, malignancies, and AIDS/HIV was calculated as previously described [25]. The length of hospital stay was described as beginning days and we included both the patients who were discharged alive and who died during admission. Seasons were defined as winter: December-February; spring: March-May; summer June-August; autumn: September-November.

Patient characteristics were analyzed using Chi-squared and independent samples t-tests as appropriate. Independent samples t-test, one-way analysis of variance and linear model were used to analyze the factors associated with LOS. In pairwise comparisons between groups Bonferroni method was used. Standardized logarithmically transformed values for LOS were used in statistical analyses due to skewness. All-cause cumulative 1-, 5- and 10-year mortality was studied with Cox regression and in-hospital mortality with logistic regression. Variables with significance level <0.1 in univariable analysis were included in multivariable models. Results are expressed as mean difference with 95% confidence interval (CI), hazard ratio (HR) with 95% confidence interval and odds ratio (OR) with 95% confidence interval. Overall 1-, 5- and 10-year mortality was determined using Kaplan-Meier estimates. Survival curves were drawn using the Kaplan-Meier method. P-values less than 0.05 were considered statistically significant. All p-values were two-sided. Statistical analyses were performed with SPSS version 25 (Armonk, NY: IBM Corp.).

## Results

We included 2166 patients in our study and 67.8% were male. The mean age of the patients was 60.7 years (SD 18.2, range 18–97). Women were older than men (mean age 63.3, SD 20.3 vs. 59.5, SD 17.0, p<0.001). There was no significant difference in CCI score between the sexes (p = 0.078). Of the patients, 49.1% were treated in the university hospital. Within 1 year prior to the IE admission, a prosthetic valve was implanted in 5.3% of the patients and a pacemaker was implanted or a pacemaker generator changed in 1.6%.

The median LOS of all the patients was 20.0 (IQR 10.0–34.0) days. It was significantly longer in men compared to women (20.0 vs. 18.0 days) (p = 0.015) (Table 1). In the youngest patients (18–39 years) the median LOS was significantly longer than in the oldest patients (24.0 vs. 16.0). The patients with CCI score of 0 had significantly longer admissions in univariable analysis compared to the patients with CCI score of ≥1. However, when adjusted for age and sex, there was no significant difference in pairwise comparisons. The length of admission was similar between the study periods 2005–2009 and 2010–2014. Furthermore, no seasonal variation was found in LOS. The median LOS in patients who died during hospitalization (n = 217) was significantly shorter, 12.0 (IQR 7.0–22.0) days, than in patients who survived (n = 1949), 20.0 (IQR 11.0–35.0) days (p<0.001).

**Table 1:** Predictors of length of stay in patients with infective endocarditis admission during 2005–2014 in Finland (n = 2166).

			Univariable model		Multivariable model^a^	
Parameter	N	Median (IQR) hospital stay (days)	Mean difference^b^ (95% CI)	P	Adjusted mean difference^b^ (95% CI)	P
Sex						
Female	697	18.0 (10.0, 31.0)	Reference		Reference	
Male	1469	20.0 (10.0, 35.0)	0.12 (0.03, 0.21)	0.009	0.11 (0.02, 0.21)	0.015
Age group				0.002		0.014
18–39 years	351	24.0 (12.0, 40.0)	Reference		Reference	
40–59 years	551	21.0 (10.0, 34.5)	-0.17 (-0.35, 0.01)	0.070	-0.18 (-0.36, 0.01)	0.067
60–79 years	933	19.0 (10.0, 32.0)	-0.16 (-0.33, 0.00)	0.055	-0.13 (-0.30, 0.04)	0.239
≥80 years	331	16.0 (9.0, 29.0)	-0.29 (-0.49, -0.09)	0.001	-0.24 (-0.44, -0.03)	0.014
Charlson Comorbidity Index (CCI) score				0.004		0.030
0	1286	21.0 (11.0, 35.0)	Reference		Reference	
1	473	17.0 (9.0, 32.0)	-0.15 (-0.27, -0.02)	0.020	-0.12 (-0.25, 0.01)	0.073
≥2	407	18.0 (9.0, 31.0)	-0.14 (-0.28, -0.01)	0.033	-0.11 (-0.25, 0.03)	0.148
Prosthetic valve implantation^c^						
Yes	115	20.0 (10.0, 34.0)	Reference			
No	2051	20.0 (10.0, 34.0)	0.01 (-0.18, 0.19)	0.962	-	-
Pacemaker operation^c^						
Yes	35	18.0 (8.5, 28.0)	Reference			
No	2131	20.0 (10.0, 34.0)	0.15 (-0.19, 0.48)	0.395	-	-
Study period						
2005–2009	1043	19.0 (10.0, 35.0)	Reference			
2010–2014	1123	20.0 (11.0, 33.0)	0.02 (-0.07, 0.10)	0.726	-	-
Season				0.829		-
Winter	545	20.0 (11.0, 34.0)	Reference			
Spring	549	20.0 (10.0, 35.0)	-0.03 (-0.19, 0.13)	1.000	-	-
Summer	552	19.0 (10.0, 32.0)	-0.06 (-0.22, 0.10)	1.000	-	-
Autumn	520	19.0 (10.0, 34.0)	-0.03 (-0.19, 0.14)	1.000	-	-

The all-cause in-hospital mortality was 10% and it was similar between men and women (9.3% vs. 11.5%, p = 0.120). The in-hospital mortality increased significantly with aging and comorbidity burden (CCI) (Table 2). Pacemaker operation or prosthetic valve implantation 1 year prior to IE admission did not affect in-hospital mortality. In-hospital mortality did not change significantly between the study periods and no seasonal variation was found.

**Table 2:** Predictors of in-hospital and 1-year mortality after infective endocarditis admission during 2005–2014 in Finland (n = 2166).

		In-hospital mortality				1-year mortality			
Parameter	N	Univariable model		Multivariable model^a^		Univariable model		Multivariable model^a^	
		OR (95% CI)	P	OR (95% CI)	P	HR (95% CI)	P	HR (95% CI)	P
Sex									
Male	1469	Reference		Reference		Reference		Reference	
Female	697	1.26 (0.94, 1.69)	0.120	-	-	1.36 (1.13, 1.63)	0.001	1.19 (0.98, 1.43)	0.075
Age group			<0.001		0.006		<0.001		<0.001
18–39 years	351	Reference		Reference		Reference		Reference	
40–59 years	551	1.73 (0.96, 3.12)	0.070	1.47 (0.81, 2.68)	0.203	2.03 (1.33, 3.11)	0.001	1.80 (1.17, 2.76)	0.008
60–79 years	933	3.00 (1.75, 5.14)	<0.001	2.24 (1.29, 3.89)	0.004	3.78 (2.56, 5.59)	<0.001	2.85 (1.92, 4.24)	<0.001
≥80 years	331	3.04 (1.68, 5.53)	<0.001	2.35 (1.28, 4.32)	0.006	5.54 (3.68, 8.34)	<0.001	4.22 (2.79, 6.39)	<0.001
Charlson Comorbidity Index (CCI) score			<0.001		<0.001		<0.001		<0.001
0	1286	Reference		Reference		Reference		Reference	
1	473	2.20 (1.56, 3.11)	<0.001	1.97 (1.39, 2.80)	<0.001	2.38 (1.92, 2.96)	<0.001	2.06 (1.65, 2.56)	<0.001
≥2	407	2.97 (2.12, 4.17)	<0.001	2.56 (1.81, 3.63)	<0.001	3.14 (2.54, 3.88)	<0.001	2.63 (2.12, 3.26)	<0.001
Prosthetic valve implantation^b^									
No	2051	Reference				Reference			
Yes	115	0.95 (0.50, 1.79)	0.868	-	-	0.74 (0.47, 1.15)	0.183	-	-
Pacemaker operation^b^									
No	2131	Reference				Reference			
Yes	35	0.84 (0.26, 2.77)	0.774	-	-	1.14 (0.59, 2.19)	0.708	-	-
Season			0.713		-		0.199		-
Winter	545	Reference				Reference			
Spring	549	1.08 (0.72, 1.61)	0.712	-	-	1.16 (0.90, 1.49)	0.264	-	-
Summer	552	1.23 (0.83, 1.81)	0.308	-	-	1.30 (1.01, 1.66)	0.041	-	-
Autumn	520	1.01 (0.67, 1.52)	0.971	-	-	1.07 (0.83, 1.40)	0.592	-	-

**Fig 1:**
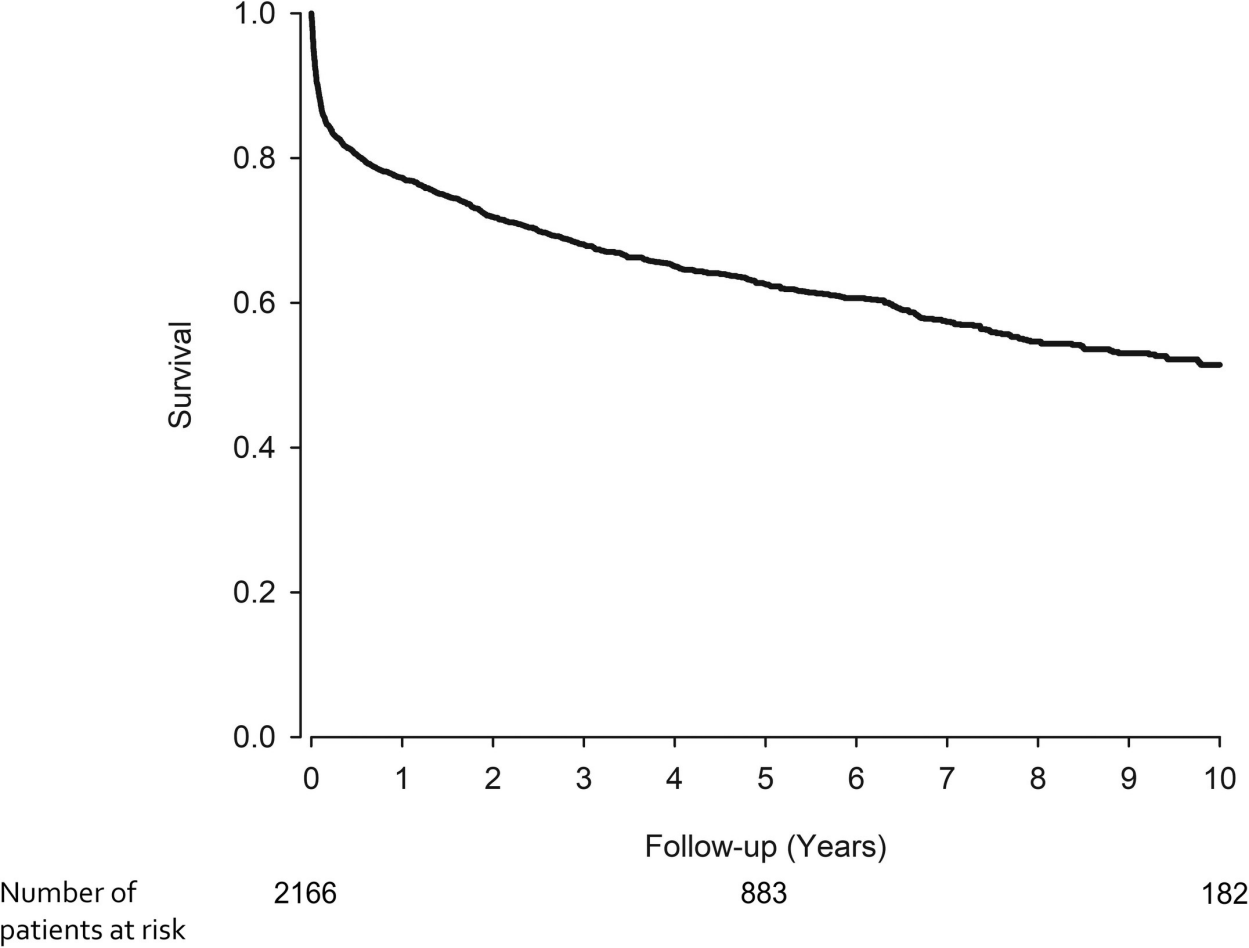
Overall 10-year survival of patients with infective endocarditis admission during 2005–2014 in Finland.

**Fig 2:**
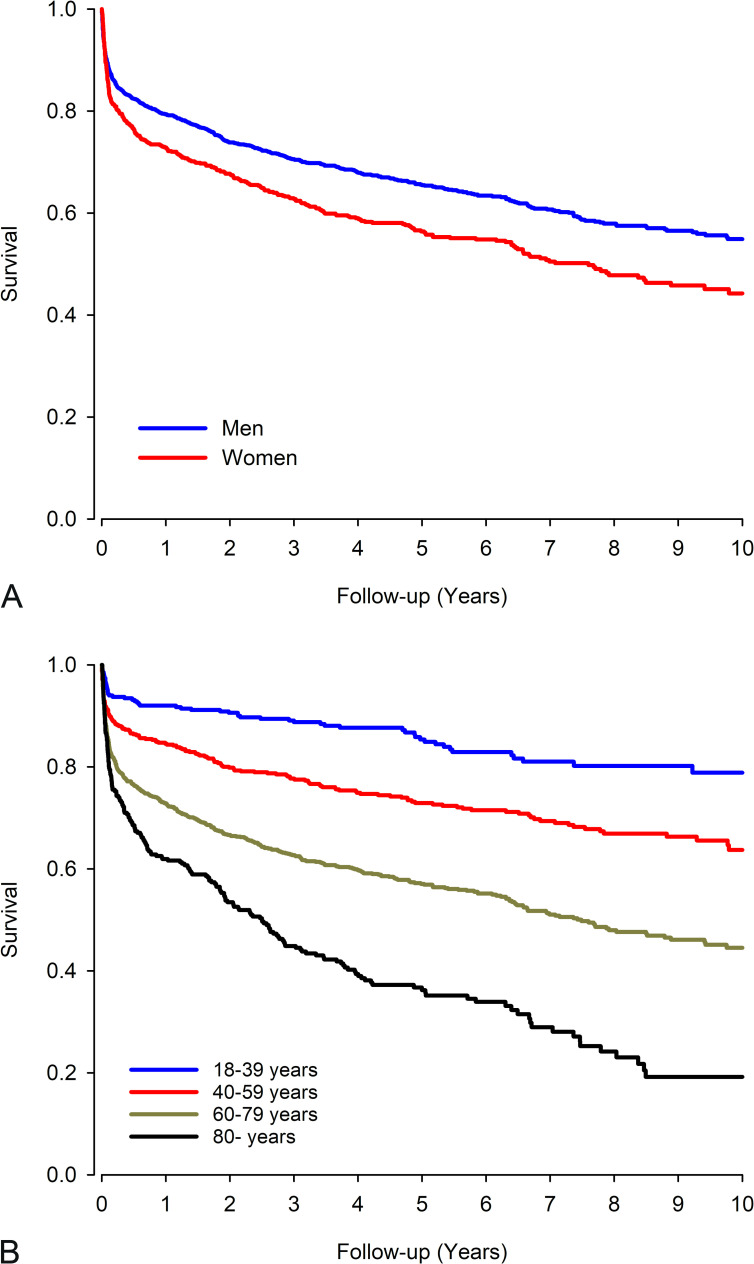
Ten-year survival of patients with infective endocarditis admission during 2005–2014 in Finland by A) sex B) age group.

The all-cause mortality was 22.7% at 1 year whereas 5- and 10-year all-cause mortality was 37.5% and 48.5%, respectively (Fig 1). Median follow-up time after IE admission was 4.0 years (range 0.0–10.0 years) for all patients, and 5.9 years (range 2.0–10.0 years) for those who survived for 10 years and 0.58 years (range 0.0–9.8 years) for those who did not. In unadjusted analysis, 1-year mortality was higher in women compared to men (27.1% vs. 20.6%, p = 0.001) (Table 2). However, in multivariable analysis (adjusted for age and CCI score) there was no difference between sexes (p = 0.075). Five- and 10-year mortality was higher in women compared to men (43.6% vs. 34.5% and 55.7% vs. 45.1%) (Table 3, Fig 2, S1 Table). The long-term mortality increased significantly with aging (Fig 2, S1 Table) and comorbidity burden (CCI) and was similar regardless of pacemaker operation or prosthetic valve implantation 1 year prior to IE admission. The 1-year mortality was stable between the study periods. Patients admitted to the hospital due to IE in summer had higher 1-year mortality compared to patients admitted in winter (HR 1.30, p = 0.041). However, in the overall comparison between the seasons there was no difference (p = 0.199). No seasonal variation was found in 5- and 10-year mortality. The underlying causes of deaths of deceased IE patients are listed in Table 4.

**Table 3:** Predictors of 5-year and 10-year mortality after infective endocarditis admission during 2005–2014 in Finland (n = 2166).

		5-year mortality				10-year mortality			
Parameter	N	Univariable model HR (95% CI)	P	Multivariable model^a^ HR (95% CI)	P	Univariable model HR (95% CI)	P	Multivariable model^a^ HR (95% CI)	P
Sex									
Male	1469	Reference		Reference		Reference		Reference	
Female	697	1.35 (1.17, 1.56)	<0.001	1.18 (1.01, 1.36)	0.034	1.35 (1.17, 1.54)	<0.001	1.18 (1.03, 1.36)	0.021
Age group			<0.001		<0.001		<0.001		<0.001
18–39 years	351	Reference		Reference		Reference		Reference	
40–59 years	551	2.08 (1.49, 2.89)	<0.001	1.88 (1.35, 2.62)	<0.001	1.94 (1.44, 2.62)	<0.001	1.79 (1.32, 2.43)	<0.001
60–79 years	933	3.72 (2.75, 5.04)	<0.001	2.90 (2.14, 3.95)	<0.001	3.54 (2.68, 4.66)	<0.001	2.85 (2.16, 3.78)	<0.001
≥80 years	331	6.21 (4.51, 8.53)	<0.001	4.96 (3.60, 6.84)	<0.001	6.10 (4.56, 8.17)	<0.001	5.00 (3.72, 6.71)	<0.001
Charlson Comorbidity Index (CCI) score			<0.001		<0.001		<0.001		<0.001
0	1286	Reference		Reference		Reference		Reference	
1	473	1.99 (1.67, 2.37)	<0.001	1.72 (1.44, 2.05)	<0.001	1.84 (1.56, 2.17)	<0.001	1.60 (1.35, 1.88)	<0.001
≥2	407	3.14 (2.66, 3.71)	<0.001	2.64 (2.23, 3.12)	<0.001	2.96 (2.53, 3.47)	<0.001	2.47 (2.11, 2.91)	<0.001
Prosthetic valve implantation^b^									
No	2051	Reference				Reference			
Yes	115	0.75 (0.53, 1.06)	0.101	-	-	0.80 (0.58, 1.09)	0.160	-	-
Pacemaker operation^b^									
No	2131	Reference				Reference			
Yes	35	1.35 (0.82, 2.21)	0.240	-	-	1.26 (0.77, 2.06)	0.363	-	-
Season			0.754		-		0.480		-
Winter	545	Reference				Reference			
Spring	549	1.09 (0.89, 1.32)	0.408	-	-	1.13 (0.94, 1.35)	0.203	-	-
Summer	552	1.02 (0.83, 1.24)	0.866	-	-	1.06 (0.88, 1.28)	0.536	-	-
Autumn	520	0.98 (0.80, 1.20)	0.835	-	-	0.99 (0.81, 1.20)	0.888	-	-

CCI = Charlson Comorbidity Index

CI = Confidence interval

HR = Hazard ratio

a Adjusted for other variables included in the multivariable model.

b Within one year prior to admission.

**Table 4:** Underlying causes of deaths in infective endocarditis patients that died within 10 years after the admission (n = 878).

Underlying cause of death	N	%
Endocarditis	153	17.4
Septicemia	60	6.8
Other infection	28	3.2
Cardiovascular or circulatory disease^a^	329	37.5
Neoplasm or blood disease^a^	110	12.5
Digestive tract disease^a^	45	5.1
Accidents or violence	39	4.4
Endocrinological disease^a^	34	3.9
Psychiatric disease	22	2.5
Nervous system disease^a^	14	1.6
Congenital malformations	12	1.4
Musculoskeletal or connective tissue disease^a^	11	1.3
Genitourinary disease^a^	11	1.3
Respiratory tract disease^a^	7	0.8
Skin or subcutaneous tissue disease^a^	1	0.1
Unspecified	2	0.2

a Excluding infections

## Discussion

In this population-based nationwide study we investigated age and sex differences, temporal trends, and factors affecting LOS due to IE and 30-day, 1-year, 5-year and 10-year mortality of IE. We found the median LOS due to IE to be 20.0 days while it varied significantly in previous studies from different countries [5,8,14,18–20]. A study from Sweden found the median LOS to be similar to ours, 23 days [2]. Substantially shorter median LOS was found in two different studies from the US: 7 days [20] and 10 days [19]. A study from France, however, found median LOS to be 43 days [5]. One reason for the shorter hospitalizations in some countries might be the wider utilization of outpatient clinics and quick transfer to other healthcare facilities. Furthermore, we found LOS significantly shorter in patients who died during the hospitalization. Comparable findings were demonstrated in studies from France and Spain [5,22]. These results suggest that if the LOS of only survived patients is studied, the hospitalizations are likely longer. However, the US study of thirty-day readmissions after IE included only patients who survived until hospital discharge and found the median LOS to be only 10 days [19].

According to our study LOS due to IE has not decreased over time. A previous study from Italy found that the median LOS increased from 30 to 35 days in the period from 2000 to 2008 [8]. Considering the financial issues, the urge to shorten hospitalizations is obvious. The introduction of the new regimen including partial oral antibiotic treatment instead of the full intravenous treatment might shorten LOS in the future [26]. However, due to the often severe course of disease, shortening the LOS with IE may be challenging.

Previous data on factors associated with LOS due to IE are scarce. Interestingly, we found that the youngest patients had significantly longer hospitalizations compared to the oldest patients. One possible reason might be that younger IE patients are more often intravenous drug users (IVDU) [27–29] and IVDUs’ IE admissions have been found to be longer than non-IVDUs’ admissions [20,27]. Intravenous drug users’ IE is more frequently caused by *S*. *aureus*, [11,28] often causing distant foci potentially requiring surgical intervention and consequently longer treatments [30,31]. Furthermore, previous studies have found surgically treated IE patients being younger [10,32–34] and having longer admissions compared to the only medically treated patients [20,21]. In addition, older patients are more commonly transferred to other healthcare facilities, e.g. health center wards, to continue with the treatment of IE. One study of IE patients between 1987 and 1996 found no difference in LOS due to IE between different age groups [35]. However, in recent years, no other studies have been conducted with IE patients on the association of age with LOS.

We found the in-hospital mortality to be 10% and it remained stable over the study periods. In previous studies the in-hospital mortality has been 6–25% [3,6,17–19,36] and it has not changed significantly over the last years [3,4,6]. We found the 1-year and 5-year mortality to be 22.7% and 37.5%, respectively. Previous studies have found 1-year mortality to be 25–37% [6–8,17] and 5-year mortality 41–53% [7,37]. In our study, the 1-year mortality remained stable over time. Previously both stable and increasing 1-year mortality have been found [6,8]. The reason for non-decreasing mortality over the years is unclear. The possibility to diagnose and treat more critically ill patients due to advancements in treatment and diagnostic methods might account for the stable mortality.

Modern and population-based data on over 5-year mortality after IE admission is scarce. We found the 10-year mortality after IE admission to be 48.5%. A study of IE patients treated in a university hospital in Finland between 1980 and 2004 found the overall survival to be 62% at 5 and 49% at 10 years after IE admission [12]. Furthermore, a study from Switzerland in a tertiary referral center between 1980 and 1995 found mortality of 42% and 50% at 5 and 10 years [13]. Accordingly, the longer time mortalities in our study have remained comparable to those in previous studies decades ago. In our previous study we found the incidence of IE to be increasing in young adults in Finland [23]. We speculated that one plausible reason might be the increasing drug abuse among young Finnish adults [38,39]. In the current study increased long-term mortality of the often young group of patients who are IVDUs might contribute to the overall long-term mortality of IE.

We found the 5- and 10-year mortality after IE admission to be higher in women compared to men. However, the in-hospital mortality was similar between the sexes and furthermore so was the 1-year mortality when adjusted for age and CCI score. Previous studies have reported similar short-term [5,10,11] and longer-term mortality [9,12,13] in women compared to men. However, a Spanish study found female sex to be an independent predictor of in-hospital mortality [14], whereas a study from UK found men to have significantly higher risk of dying during the IE admission [16]. Long-term mortality was found to be higher in women in a previous study from the Netherlands [15]. Mortality differences between the sexes need to be further examined.

No seasonal variation in in-hospital, 5- or 10-year mortality or LOS was found. These results suggest that the severity of IE is not associated with the time of admission. Furthermore, IE is a serious disease and, for example, the holiday periods with limited experienced health care staff or temporarily closed wards with fewer beds available overall do not affect the quality of care of IE or LOS. Previously, our study of 754 IE-related deaths from Finland during 2004–2016 found no seasonal difference in the occurrence of fatal IE [40]. Moreover, another study from Finland found no difference in LOS or mortality during internal medicine ward admissions between July and November [41]. Previously, seasonal variation in mortality has been found in sepsis [42], however data on seasonality of IE is limited. A study from France from 2003 to 2015 found that the incidence of cardiac implantable electronic device infections was positively associated with high temperature and precipitation [43].

Our study has some limitations. Our data is register data and was retrospectively collected. The diagnoses were made by clinicians and we did not have access to detailed clinical patient data. We did not have the microbiological or other laboratory data or data on the possible complications of IE, which limits our results. Especially microbiological etiology would have been of interest, and in particular *S*. *aureus*, as it has been described to be a risk factor for mortality in IE [4–6,14,17]. To assess the burden of possible risk factors, we used the CCI score [25]. Unfortunately, we did not have the information of the possible hemodialysis, which has been reported to be associated with increased mortality in IE [44–46]. Additionally, some of the diagnoses might be missing and there might be errors in coding. However, the accuracy of the obligatory and nationwide CRHC has been found to be precise [47]. Furthermore, in previous diagnosis validation testing we found the specificity of the ICD-10 codes for definite or possible IE according to Duke criteria to be remarkably high, 96.8% [23]. However, our current study included 38 hospitals and there might be some variation in the consistency of ICD-10 coding between the hospitals. Accordingly, a previous Canadian study on the validation of ICD-10 codes for IE reported excellent specificity (100%) and very good (90%) sensitivity [48]. The strength of our study is that it is a large population-based study covering all hospitals treating patients with IE in Finland. Moreover, our study provides novel information on the factors associated with LOS and 10-year mortality of IE.

In conclusion, according to our study men had longer hospital stays due to IE compared to women. The 5- and 10-year mortality was higher in women and mortality increased with aging and comorbidity burden. The mortality of IE or LOS did not change over time.

## Supporting information

S1 Table Number of patients at risk in Fig 2 displaying 10-year survival of patients with infective endocarditis admission during 2005–2014 in Finland by A) sex B) age group.
(DOCX)
