# Supplementary material for: Short- and long-term outcomes of infective endocarditis admission in adults: A population-based registry study in Finland
Source: PLoS One. 2021 Jul 15;16(7):e0254553. doi: 10.1371/journal.pone.0254553 (PMC8282023; doi:10.1371/journal.pone.0254553)
Supplement: S1 Table — Number of patients at risk in Fig 2 displaying 10-year survival of patients with infective endocarditis admission during 2005–2014 in Finland by A) sex B) age group. (DOCX) [file pone.0254553.s001.docx]

|  | | | Number of patients at risk | | |
| --- | --- | --- | --- | --- | --- |
|  | | | At 0 years | At 5 years | At 10 years |
| Sex | | |  |  |  |
|  | | Men | 1469 | 618 | 137 |
|  | | Women | 697 | 265 | 45 |
| Age group | | |  |  |  |
|  | 18-39 years | | 351 | 181 | 45 |
|  | 40-59 years | | 551 | 273 | 62 |
|  | 60-79 years | | 933 | 361 | 68 |
|  | ≥80 years | | 331 | 68 | 7 |
|  | | |  |  |  |
